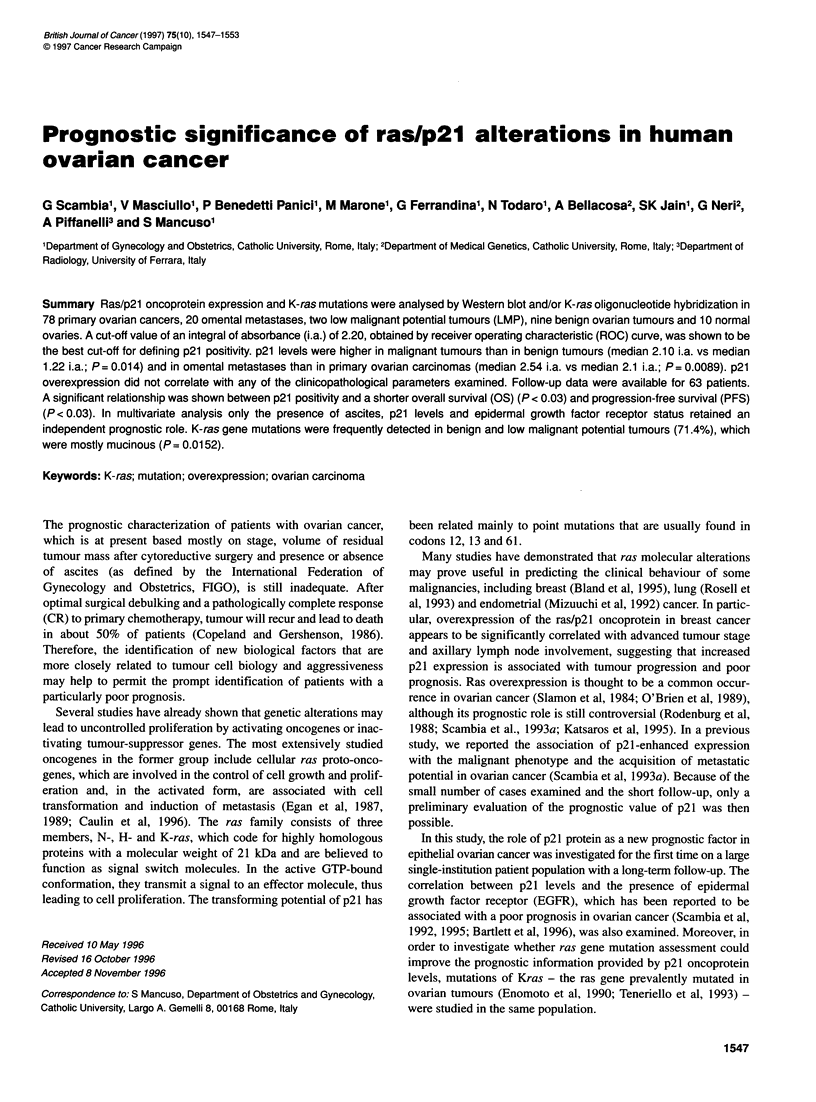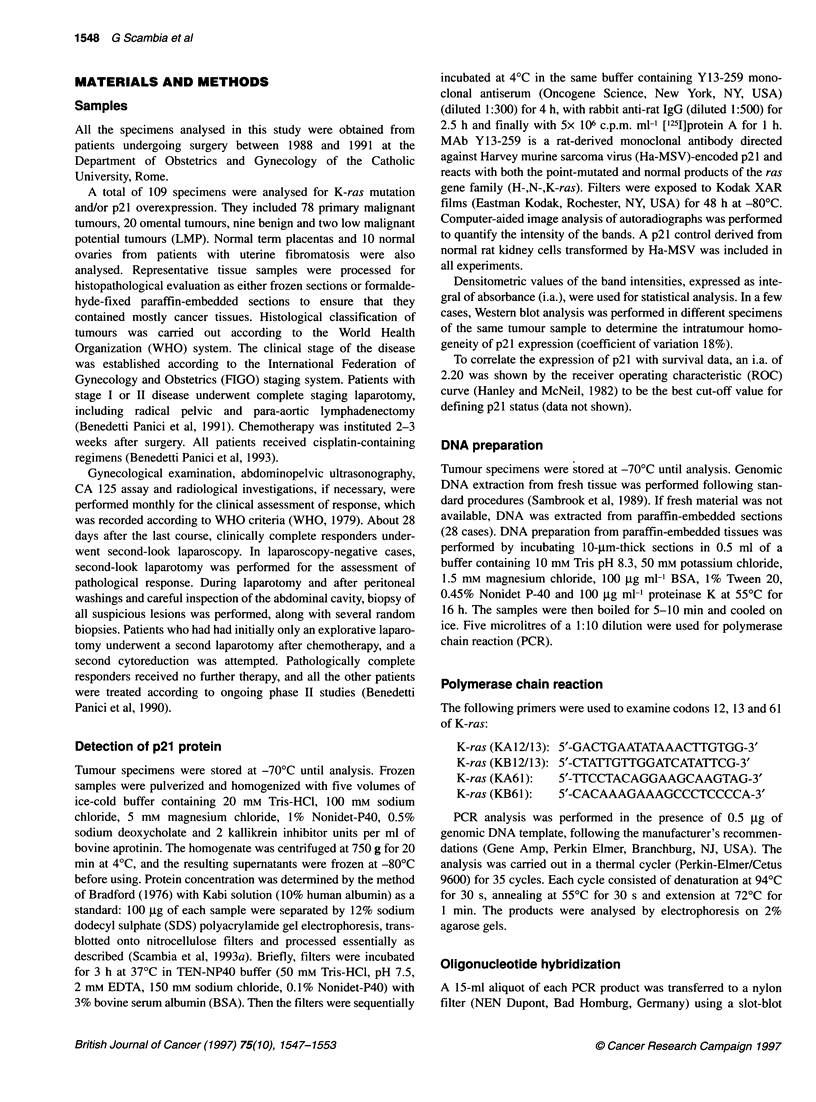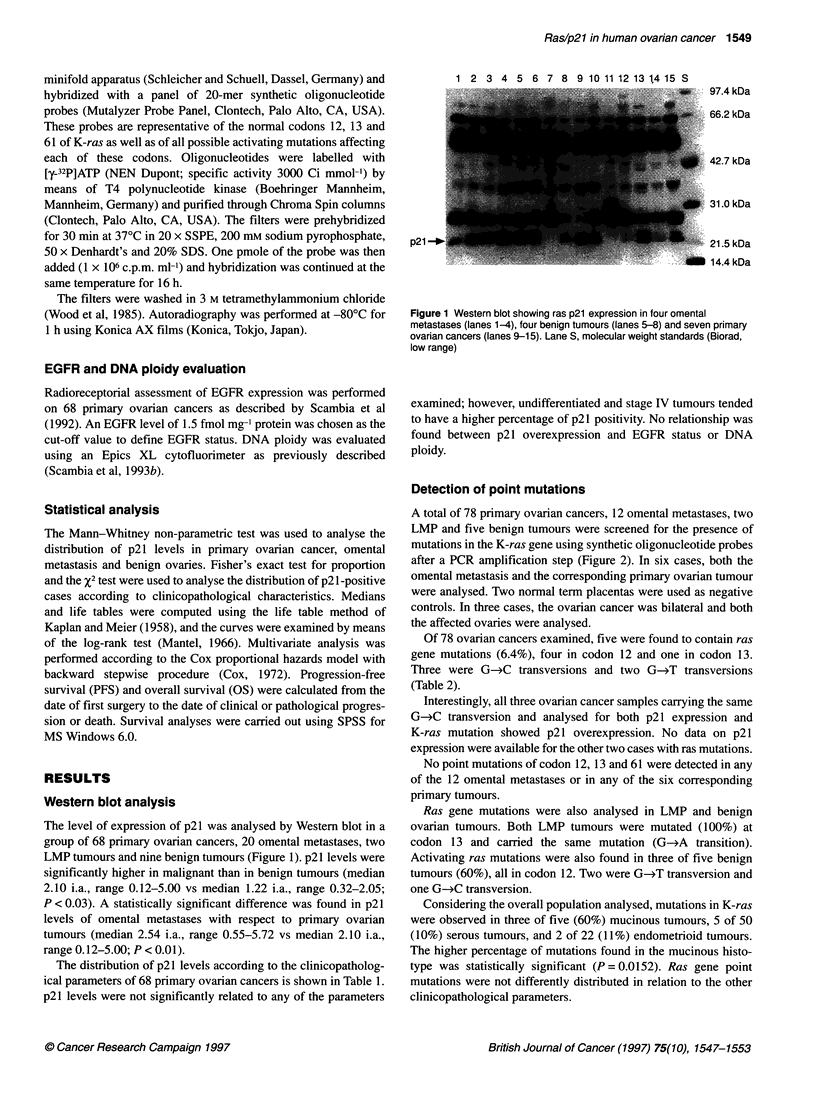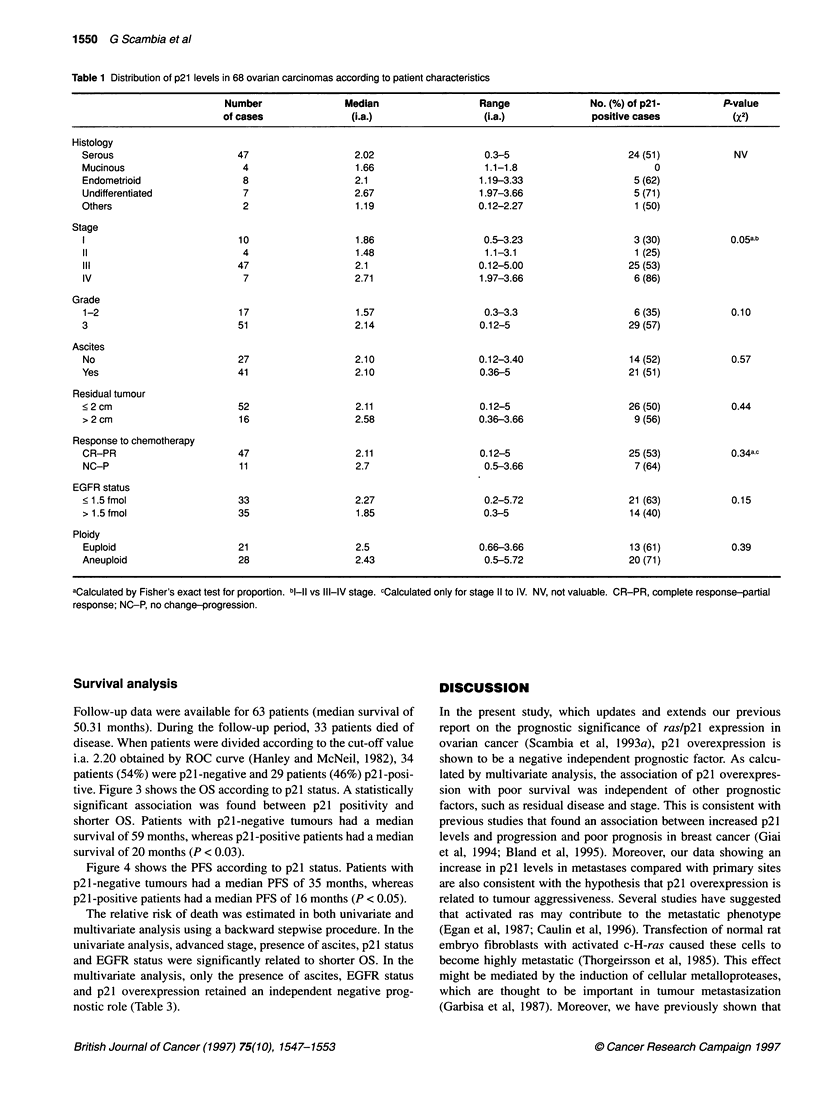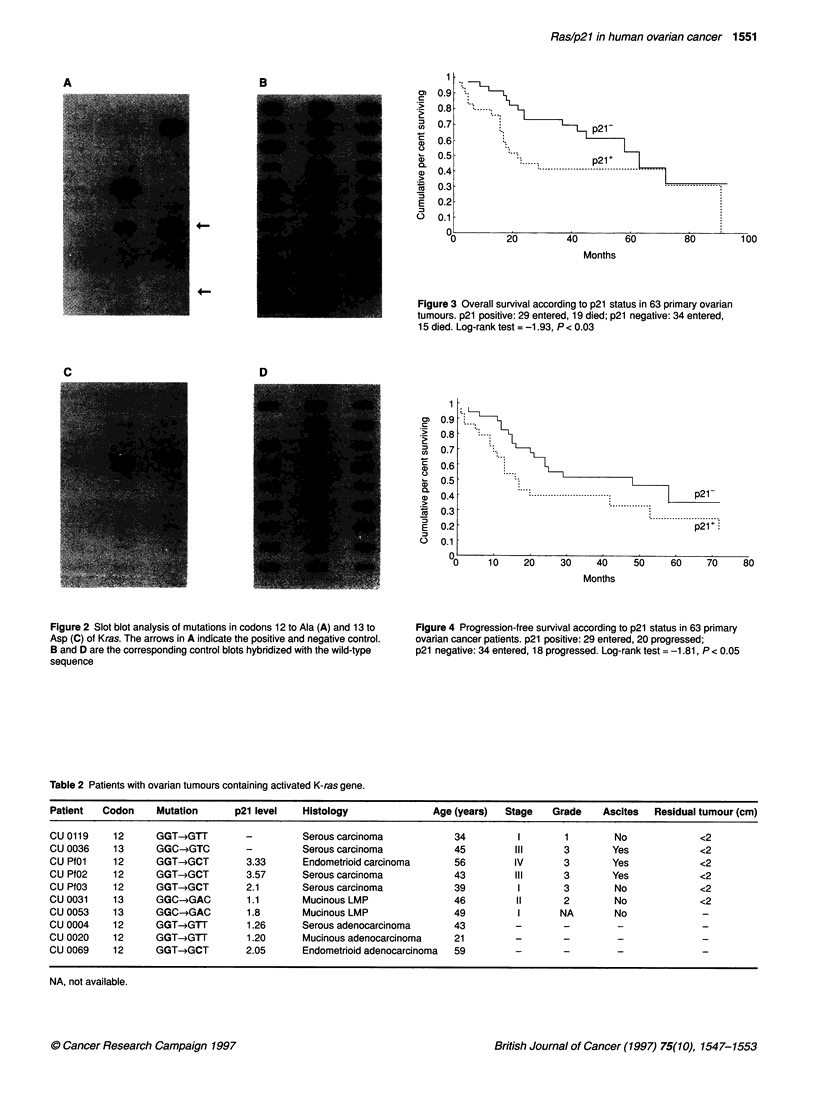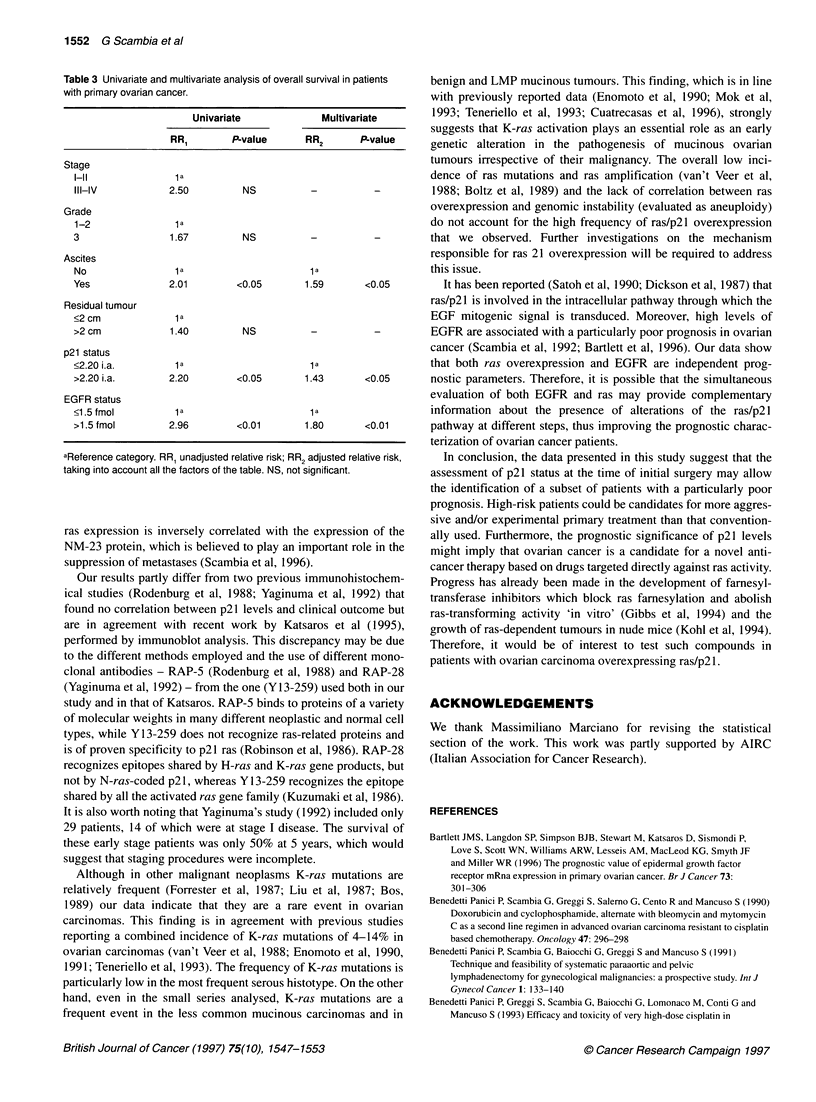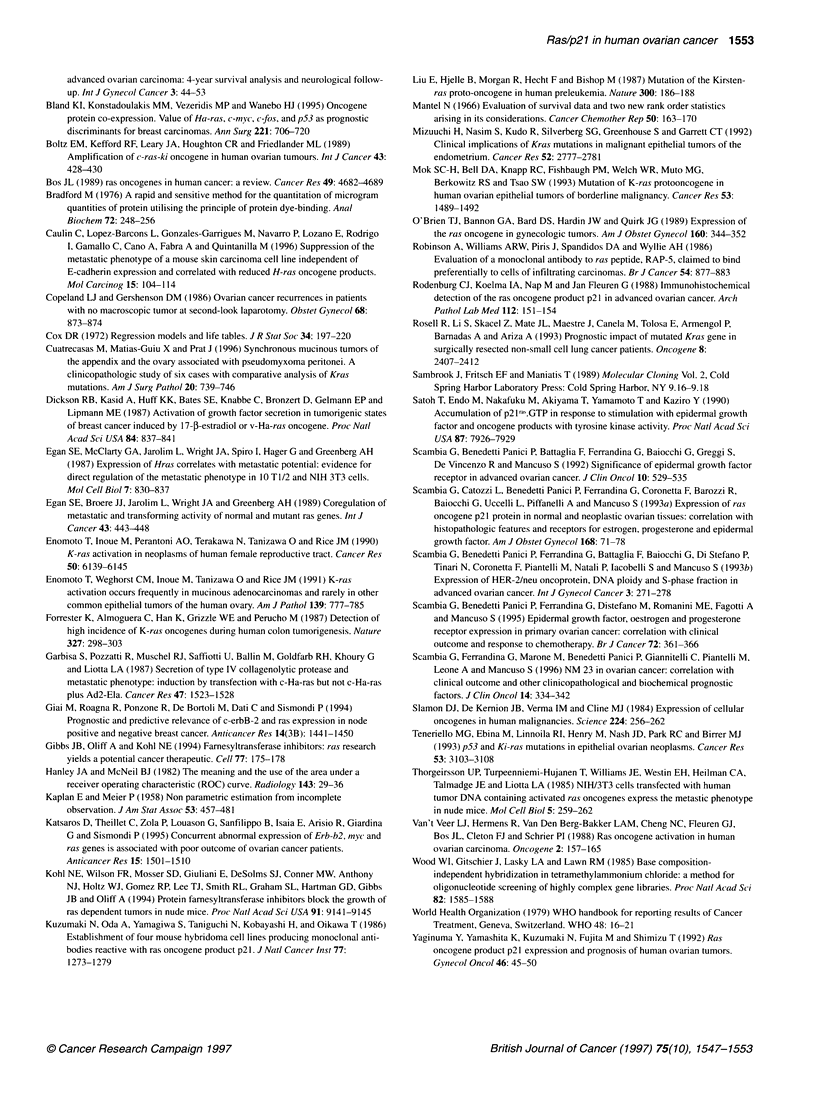# Prognostic significance of ras/p21 alterations in human ovarian cancer.

**DOI:** 10.1038/bjc.1997.264

**Published:** 1997

**Authors:** G. Scambia, V. Masciullo, P. Benedetti Panici, M. Marone, G. Ferrandina, N. Todaro, A. Bellacosa, S. K. Jain, G. Neri, A. Piffanelli, S. Mancuso

**Affiliations:** Department of Gynecology and Obstetrics, Catholic University, Rome, Italy.

## Abstract

**Images:**


British Journal of Cancer (1997) 75(10), 1547–1553
© 1997 Cancer Research Campaign

### Prognostic significance of ras/p21 alterations in human ovarian cancer

**G Scambia[1], V Masciullo[1], P Benedetti Panici[1], M Marone[1], G Ferrandina[1], N Todaro[1], A Bellacosa[2], SK Jain[1], G Neri[2], A Piffanelli[3] and S Mancuso[1]**

[1]Department of Gynecology and Obstetrics, Catholic University, Rome, Italy; [2]Department of Medical Genetics, Catholic University, Rome, Italy; [3]Department of Radiology, University of Ferrara, Italy

**Summary** Ras/p21 oncoprotein expression and K-*ras* mutations were analysed by Western blot and/or K-*ras* oligonucleotide hybridization in 78 primary ovarian cancers, 20 omental metastases, two low malignant potential tumours (LMP), nine benign ovarian tumours and 10 normal ovaries. A cut-off value of an integral of absorbance (i.a.) of 2.20, obtained by receiver operating characteristic (ROC) curve, was shown to be the best cut-off for defining p21 positivity. p21 levels were higher in malignant tumours than in benign tumours (median 2.10 i.a. vs median 1.22 i.a.; $P = 0.014$) and in omental metastases than in primary ovarian carcinomas (median 2.54 i.a. vs median 2.1 i.a.; $P = 0.0089$). p21 overexpression did not correlate with any of the clinicopathological parameters examined. Follow-up data were available for 63 patients. A significant relationship was shown between p21 positivity and a shorter overall survival (OS) ($P < 0.03$) and progression-free survival (PFS) ($P < 0.03$). In multivariate analysis only the presence of ascites, p21 levels and epidermal growth factor receptor status retained an independent prognostic role. K-*ras* gene mutations were frequently detected in benign and low malignant potential tumours (71.4%), which were mostly mucinous ($P = 0.0152$).

**Keywords:** K-*ras*; mutation; overexpression; ovarian carcinoma

The prognostic characterization of patients with ovarian cancer, which is at present based mostly on stage, volume of residual tumour mass after cytoreductive surgery and presence or absence of ascites (as defined by the International Federation of Gynecology and Obstetrics, FIGO), is still inadequate. After optimal surgical debulking and a pathologically complete response (CR) to primary chemotherapy, tumour will recur and lead to death in about 50% of patients (Copeland and Gershenson, 1986). Therefore, the identification of new biological factors that are more closely related to tumour cell biology and aggressiveness may help to permit the prompt identification of patients with a particularly poor prognosis.

Several studies have already shown that genetic alterations may lead to uncontrolled proliferation by activating oncogenes or inactivating tumour-suppressor genes. The most extensively studied oncogenes in the former group include cellular *ras* proto-oncogenes, which are involved in the control of cell growth and proliferation and, in the activated form, are associated with cell transformation and induction of metastasis (Egan et al, 1987, 1989; Caulin et al, 1996). The *ras* family consists of three members, N-, H- and K-*ras*, which code for highly homologous proteins with a molecular weight of 21 kDa and are believed to function as signal switch molecules. In the active GTP-bound conformation, they transmit a signal to an effector molecule, thus leading to cell proliferation. The transforming potential of p21 has been related mainly to point mutations that are usually found in codons 12, 13 and 61.

Many studies have demonstrated that *ras* molecular alterations may prove useful in predicting the clinical behaviour of some malignancies, including breast (Bland et al, 1995), lung (Rosell et al, 1993) and endometrial (Mizuuchi et al, 1992) cancer. In particular, overexpression of the ras/p21 oncoprotein in breast cancer appears to be significantly correlated with advanced tumour stage and axillary lymph node involvement, suggesting that increased p21 expression is associated with tumour progression and poor prognosis. Ras overexpression is thought to be a common occurrence in ovarian cancer (Slamon et al, 1984; O'Brien et al, 1989), although its prognostic role is still controversial (Rodenburg et al, 1988; Scambia et al., 1993*a*; Katsaros et al, 1995). In a previous study, we reported the association of p21-enhanced expression with the malignant phenotype and the acquisition of metastatic potential in ovarian cancer (Scambia et al, 1993*a*). Because of the small number of cases examined and the short follow-up, only a preliminary evaluation of the prognostic value of p21 was then possible.

In this study, the role of p21 protein as a new prognostic factor in epithelial ovarian cancer was investigated for the first time on a large single-institution patient population with a long-term follow-up. The correlation between p21 levels and the presence of epidermal growth factor receptor (EGFR), which has been reported to be associated with a poor prognosis in ovarian cancer (Scambia et al, 1992, 1995; Bartlett et al, 1996), was also examined. Moreover, in order to investigate whether *ras* gene mutation assessment could improve the prognostic information provided by p21 oncoprotein levels, mutations of K*ras* – the ras gene prevalently mutated in ovarian tumours (Enomoto et al, 1990; Teneriello et al, 1993) – were studied in the same population.

*Received 10 May 1996*
*Revised 16 October 1996*
*Accepted 8 November 1996*

*Correspondence to:* S Mancuso, Department of Obstetrics and Gynecology, Catholic University, Largo A. Gemelli 8, 00168 Rome, Italy

### MATERIALS AND METHODS

### Samples

All the specimens analysed in this study were obtained from patients undergoing surgery between 1988 and 1991 at the Department of Obstetrics and Gynecology of the Catholic University, Rome.

A total of 109 specimens were analysed for K-*ras* mutation and/or p21 overexpression. They included 78 primary malignant tumours, 20 omental tumours, nine benign and two low malignant potential tumours (LMP). Normal term placentas and 10 normal ovaries from patients with uterine fibromatosis were also analysed. Representative tissue samples were processed for histopathological evaluation as either frozen sections or formaldehyde-fixed paraffin-embedded sections to ensure that they contained mostly cancer tissues. Histological classification of tumours was carried out according to the World Health Organization (WHO) system. The clinical stage of the disease was established according to the International Federation of Gynecology and Obstetrics (FIGO) staging system. Patients with stage I or II disease underwent complete staging laparotomy, including radical pelvic and para-aortic lymphadenectomy (Benedetti Panici et al, 1991). Chemotherapy was instituted 2–3 weeks after surgery. All patients received cisplatin-containing regimens (Benedetti Panici et al, 1993).

Gynecological examination, abdominopelvic ultrasonography, CA 125 assay and radiological investigations, if necessary, were performed monthly for the clinical assessment of response, which was recorded according to WHO criteria (WHO, 1979). About 28 days after the last course, clinically complete responders underwent second-look laparoscopy. In laparoscopy-negative cases, second-look laparotomy was performed for the assessment of pathological response. During laparotomy and after peritoneal washings and careful inspection of the abdominal cavity, biopsy of all suspicious lesions was performed, along with several random biopsies. Patients who had had initially only an explorative laparotomy underwent a second laparotomy after chemotherapy, and a second cytoreduction was attempted. Pathologically complete responders received no further therapy, and all the other patients were treated according to ongoing phase II studies (Benedetti Panici et al, 1990).

### Detection of p21 protein

Tumour specimens were stored at –70°C until analysis. Frozen samples were pulverized and homogenized with five volumes of ice-cold buffer containing 20 mM Tris-HCl, 100 mM sodium chloride, 5 mM magnesium chloride, 1% Nonidet-P40, 0.5% sodium deoxycholate and 2 kallikrein inhibitor units per ml of bovine aprotinin. The homogenate was centrifuged at 750 **g** for 20 min at 4°C, and the resulting supernatants were frozen at –80°C before using. Protein concentration was determined by the method of Bradford (1976) with Kabi solution (10% human albumin) as a standard: 100 μg of each sample were separated by 12% sodium dodecyl sulphate (SDS) polyacrylamide gel electrophoresis, transblotted onto nitrocellulose filters and processed essentially as described (Scambia et al, 1993*a*). Briefly, filters were incubated for 3 h at 37°C in TEN-NP40 buffer (50 mM Tris-HCl, pH 7.5, 2 mM EDTA, 150 mM sodium chloride, 0.1% Nonidet-P40) with 3% bovine serum albumin (BSA). Then the filters were sequentially incubated at 4°C in the same buffer containing Y13-259 monoclonal antiserum (Oncogene Science, New York, NY, USA) (diluted 1:300) for 4 h, with rabbit anti-rat IgG (diluted 1:500) for 2.5 h and finally with 5× $10^6$ c.p.m. $ml^{-1}$ [$^{125}$I]protein A for 1 h. MAb Y13-259 is a rat-derived monoclonal antibody directed against Harvey murine sarcoma virus (Ha-MSV)-encoded p21 and reacts with both the point-mutated and normal products of the *ras* gene family (H-,N-,K-*ras*). Filters were exposed to Kodak XAR films (Eastman Kodak, Rochester, NY, USA) for 48 h at –80°C. Computer-aided image analysis of autoradiographs was performed to quantify the intensity of the bands. A p21 control derived from normal rat kidney cells transformed by Ha-MSV was included in all experiments.

Densitometric values of the band intensities, expressed as integral of absorbance (i.a.), were used for statistical analysis. In a few cases, Western blot analysis was performed in different specimens of the same tumour sample to determine the intratumour homogeneity of p21 expression (coefficient of variation 18%).

To correlate the expression of p21 with survival data, an i.a. of 2.20 was shown by the receiver operating characteristic (ROC) curve (Hanley and McNeil, 1982) to be the best cut-off value for defining p21 status (data not shown).

### DNA preparation

Tumour specimens were stored at –70°C until analysis. Genomic DNA extraction from fresh tissue was performed following standard procedures (Sambrook et al, 1989). If fresh material was not available, DNA was extracted from paraffin-embedded sections (28 cases). DNA preparation from paraffin-embedded tissues was performed by incubating 10-μm-thick sections in 0.5 ml of a buffer containing 10 mM Tris pH 8.3, 50 mM potassium chloride, 1.5 mM magnesium chloride, 100 μg $ml^{-1}$ BSA, 1% Tween 20, 0.45% Nonidet P-40 and 100 μg $ml^{-1}$ proteinase K at 55°C for 16 h. The samples were then boiled for 5–10 min and cooled on ice. Five microlitres of a 1:10 dilution were used for polymerase chain reaction (PCR).

### Polymerase chain reaction

The following primers were used to examine codons 12, 13 and 61 of K-*ras*:

| | |
|---|---|
| K-*ras* (KA12/13): | 5′-GACTGAATATAAACTTGTGG-3′ |
| K-*ras* (KB12/13): | 5′-CTATTGTTGGATCATATTCG-3′ |
| K-*ras* (KA61): | 5′-TTCCTACAGGAAGCAAGTAG-3′ |
| K-*ras* (KB61): | 5′-CACAAAGAAAGCCCTCCCCA-3′ |

PCR analysis was performed in the presence of 0.5 μg of genomic DNA template, following the manufacturer's recommendations (Gene Amp, Perkin Elmer, Branchburg, NJ, USA). The analysis was carried out in a thermal cycler (Perkin-Elmer/Cetus 9600) for 35 cycles. Each cycle consisted of denaturation at 94°C for 30 s, annealing at 55°C for 30 s and extension at 72°C for 1 min. The products were analysed by electrophoresis on 2% agarose gels.

### Oligonucleotide hybridization

A 15-ml aliquot of each PCR product was transferred to a nylon filter (NEN Dupont, Bad Homburg, Germany) using a slot-blot

© Cancer Research Campaign 1997

minifold apparatus (Schleicher and Schuell, Dassel, Germany) and hybridized with a panel of 20-mer synthetic oligonucleotide probes (Mutalyzer Probe Panel, Clontech, Palo Alto, CA, USA). These probes are representative of the normal codons 12, 13 and 61 of K-*ras* as well as of all possible activating mutations affecting each of these codons. Oligonucleotides were labelled with [$\gamma$-$^{32}$P]ATP (NEN Dupont; specific activity 3000 Ci mmol$^{-1}$) by means of T4 polynucleotide kinase (Boehringer Mannheim, Mannheim, Germany) and purified through Chroma Spin columns (Clontech, Palo Alto, CA, USA). The filters were prehybridized for 30 min at 37°C in 20 × SSPE, 200 mM sodium pyrophosphate, 50 × Denhardt's and 20% SDS. One pmole of the probe was then added (1 × 10$^6$ c.p.m. ml$^{-1}$) and hybridization was continued at the same temperature for 16 h.

The filters were washed in 3 M tetramethylammonium chloride (Wood et al, 1985). Autoradiography was performed at –80°C for 1 h using Konica AX films (Konica, Tokjo, Japan).

**Figure 1** Western blot showing ras p21 expression in four omental metastases (lanes 1–4), four benign tumours (lanes 5–8) and seven primary ovarian cancers (lanes 9–15). Lane S, molecular weight standards (Biorad, low range)

### EGFR and DNA ploidy evaluation

Radioreceptorial assessment of EGFR expression was performed on 68 primary ovarian cancers as described by Scambia et al (1992). An EGFR level of 1.5 fmol mg$^{-1}$ protein was chosen as the cut-off value to define EGFR status. DNA ploidy was evaluated using an Epics XL cytofluorimeter as previously described (Scambia et al, 1993*b*).

### Statistical analysis

The Mann–Whitney non-parametric test was used to analyse the distribution of p21 levels in primary ovarian cancer, omental metastasis and benign ovaries. Fisher's exact test for proportion and the $\chi^2$ test were used to analyse the distribution of p21-positive cases according to clinicopathological characteristics. Medians and life tables were computed using the life table method of Kaplan and Meier (1958), and the curves were examined by means of the log-rank test (Mantel, 1966). Multivariate analysis was performed according to the Cox proportional hazards model with backward stepwise procedure (Cox, 1972). Progression-free survival (PFS) and overall survival (OS) were calculated from the date of first surgery to the date of clinical or pathological progression or death. Survival analyses were carried out using SPSS for MS Windows 6.0.

### RESULTS

### Western blot analysis

The level of expression of p21 was analysed by Western blot in a group of 68 primary ovarian cancers, 20 omental metastases, two LMP tumours and nine benign tumours (Figure 1). p21 levels were significantly higher in malignant than in benign tumours (median 2.10 i.a., range 0.12–5.00 vs median 1.22 i.a., range 0.32–2.05; $P < 0.03$). A statistically significant difference was found in p21 levels of omental metastases with respect to primary ovarian tumours (median 2.54 i.a., range 0.55–5.72 vs median 2.10 i.a., range 0.12–5.00; $P < 0.01$).

The distribution of p21 levels according to the clinicopathological parameters of 68 primary ovarian cancers is shown in Table 1. p21 levels were not significantly related to any of the parameters examined; however, undifferentiated and stage IV tumours tended to have a higher percentage of p21 positivity. No relationship was found between p21 overexpression and EGFR status or DNA ploidy.

### Detection of point mutations

A total of 78 primary ovarian cancers, 12 omental metastases, two LMP and five benign tumours were screened for the presence of mutations in the K-*ras* gene using synthetic oligonucleotide probes after a PCR amplification step (Figure 2). In six cases, both the omental metastasis and the corresponding primary ovarian tumour were analysed. Two normal term placentas were used as negative controls. In three cases, the ovarian cancer was bilateral and both the affected ovaries were analysed.

Of 78 ovarian cancers examined, five were found to contain *ras* gene mutations (6.4%), four in codon 12 and one in codon 13. Three were G→C transversions and two G→T transversions (Table 2).

Interestingly, all three ovarian cancer samples carrying the same G→C transversion and analysed for both p21 expression and K-*ras* mutation showed p21 overexpression. No data on p21 expression were available for the other two cases with ras mutations.

No point mutations of codon 12, 13 and 61 were detected in any of the 12 omental metastases or in any of the six corresponding primary tumours.

*Ras* gene mutations were also analysed in LMP and benign ovarian tumours. Both LMP tumours were mutated (100%) at codon 13 and carried the same mutation (G→A transition). Activating *ras* mutations were also found in three of five benign tumours (60%), all in codon 12. Two were G→T transversion and one G→C transversion.

Considering the overall population analysed, mutations in K-*ras* were observed in three of five (60%) mucinous tumours, 5 of 50 (10%) serous tumours, and 2 of 22 (11%) endometrioid tumours. The higher percentage of mutations found in the mucinous histotype was statistically significant ($P = 0.0152$). *Ras* gene point mutations were not differently distributed in relation to the other clinicopathological parameters.

© Cancer Research Campaign 1997 

**Table 1** Distribution of p21 levels in 68 ovarian carcinomas according to patient characteristics

| | Number of cases | Median (i.a.) | Range (i.a.) | No. (%) of p21-positive cases | *P*-value ($\chi^2$) |
|---|---|---|---|---|---|
| Histology | | | | | |
| Serous | 47 | 2.02 | 0.3–5 | 24 (51) | NV |
| Mucinous | 4 | 1.66 | 1.1–1.8 | 0 | |
| Endometrioid | 8 | 2.1 | 1.19–3.33 | 5 (62) | |
| Undifferentiated | 7 | 2.67 | 1.97–3.66 | 5 (71) | |
| Others | 2 | 1.19 | 0.12–2.27 | 1 (50) | |
| Stage | | | | | |
| I | 10 | 1.86 | 0.5–3.23 | 3 (30) | 0.05[a,b] |
| II | 4 | 1.48 | 1.1–3.1 | 1 (25) | |
| III | 47 | 2.1 | 0.12–5.00 | 25 (53) | |
| IV | 7 | 2.71 | 1.97–3.66 | 6 (86) | |
| Grade | | | | | |
| 1–2 | 17 | 1.57 | 0.3–3.3 | 6 (35) | 0.10 |
| 3 | 51 | 2.14 | 0.12–5 | 29 (57) | |
| Ascites | | | | | |
| No | 27 | 2.10 | 0.12–3.40 | 14 (52) | 0.57 |
| Yes | 41 | 2.10 | 0.36–5 | 21 (51) | |
| Residual tumour | | | | | |
| ≤ 2 cm | 52 | 2.11 | 0.12–5 | 26 (50) | 0.44 |
| > 2 cm | 16 | 2.58 | 0.36–3.66 | 9 (56) | |
| Response to chemotherapy | | | | | |
| CR–PR | 47 | 2.11 | 0.12–5 | 25 (53) | 0.34[a,c] |
| NC–P | 11 | 2.7 | 0.5–3.66 | 7 (64) | |
| EGFR status | | | | | |
| ≤ 1.5 fmol | 33 | 2.27 | 0.2–5.72 | 21 (63) | 0.15 |
| > 1.5 fmol | 35 | 1.85 | 0.3–5 | 14 (40) | |
| Ploidy | | | | | |
| Euploid | 21 | 2.5 | 0.66–3.66 | 13 (61) | 0.39 |
| Aneuploid | 28 | 2.43 | 0.5–5.72 | 20 (71) | |

[a]Calculated by Fisher's exact test for proportion. [b]I–II vs III–IV stage. [c]Calculated only for stage II to IV. NV, not valuable. CR–PR, complete response–partial response; NC–P, no change–progression.

### Survival analysis

Follow-up data were available for 63 patients (median survival of 50.31 months). During the follow-up period, 33 patients died of disease. When patients were divided according to the cut-off value i.a. 2.20 obtained by ROC curve (Hanley and McNeil, 1982), 34 patients (54%) were p21-negative and 29 patients (46%) p21-positive. Figure 3 shows the OS according to p21 status. A statistically significant association was found between p21 positivity and shorter OS. Patients with p21-negative tumours had a median survival of 59 months, whereas p21-positive patients had a median survival of 20 months ($P < 0.03$).

Figure 4 shows the PFS according to p21 status. Patients with p21-negative tumours had a median PFS of 35 months, whereas p21-positive patients had a median PFS of 16 months ($P < 0.05$).

The relative risk of death was estimated in both univariate and multivariate analysis using a backward stepwise procedure. In the univariate analysis, advanced stage, presence of ascites, p21 status and EGFR status were significantly related to shorter OS. In the multivariate analysis, only the presence of ascites, EGFR status and p21 overexpression retained an independent negative prognostic role (Table 3).

### DISCUSSION

In the present study, which updates and extends our previous report on the prognostic significance of *ras*/p21 expression in ovarian cancer (Scambia et al, 1993*a*), p21 overexpression is shown to be a negative independent prognostic factor. As calculated by multivariate analysis, the association of p21 overexpression with poor survival was independent of other prognostic factors, such as residual disease and stage. This is consistent with previous studies that found an association between increased p21 levels and progression and poor prognosis in breast cancer (Giai et al, 1994; Bland et al, 1995). Moreover, our data showing an increase in p21 levels in metastases compared with primary sites are also consistent with the hypothesis that p21 overexpression is related to tumour aggressiveness. Several studies have suggested that activated ras may contribute to the metastatic phenotype (Egan et al, 1987; Caulin et al, 1996). Transfection of normal rat embryo fibroblasts with activated c-H-*ras* caused these cells to become highly metastatic (Thorgeirsson et al, 1985). This effect might be mediated by the induction of cellular metalloproteases, which are thought to be important in tumour metastasization (Garbisa et al, 1987). Moreover, we have previously shown that

 © Cancer Research Campaign 1997

**Figure 2** Slot blot analysis of mutations in codons 12 to Ala (**A**) and 13 to Asp (**C**) of K*ras*. The arrows in **A** indicate the positive and negative control. **B** and **D** are the corresponding control blots hybridized with the wild-type sequence

**Figure 3** Overall survival according to p21 status in 63 primary ovarian tumours. p21 positive: 29 entered, 19 died; p21 negative: 34 entered, 15 died. Log-rank test = −1.93, $P < 0.03$

**Figure 4** Progression-free survival according to p21 status in 63 primary ovarian cancer patients. p21 positive: 29 entered, 20 progressed; p21 negative: 34 entered, 18 progressed. Log-rank test = −1.81, $P < 0.05$

**Table 2** Patients with ovarian tumours containing activated K-*ras* gene.

| Patient | Codon | Mutation | p21 level | Histology | Age (years) | Stage | Grade | Ascites | Residual tumour (cm) |
|---|---|---|---|---|---|---|---|---|---|
| CU 0119 | 12 | GGT→GTT | – | Serous carcinoma | 34 | I | 1 | No | <2 |
| CU 0036 | 13 | GGC→GTC | – | Serous carcinoma | 45 | III | 3 | Yes | <2 |
| CU Pf01 | 12 | GGT→GCT | 3.33 | Endometrioid carcinoma | 56 | IV | 3 | Yes | <2 |
| CU Pf02 | 12 | GGT→GCT | 3.57 | Serous carcinoma | 43 | III | 3 | Yes | <2 |
| CU Pf03 | 12 | GGT→GCT | 2.1 | Serous carcinoma | 39 | I | 3 | No | <2 |
| CU 0031 | 13 | GGC→GAC | 1.1 | Mucinous LMP | 46 | II | 2 | No | <2 |
| CU 0053 | 13 | GGC→GAC | 1.8 | Mucinous LMP | 49 | I | NA | No | – |
| CU 0004 | 12 | GGT→GTT | 1.26 | Serous adenocarcinoma | 43 | – | – | – | – |
| CU 0020 | 12 | GGT→GTT | 1.20 | Mucinous adenocarcinoma | 21 | – | – | – | – |
| CU 0069 | 12 | GGT→GCT | 2.05 | Endometrioid adenocarcinoma | 59 | – | – | – | – |

NA, not available.

Table 3 Univariate and multivariate analysis of overall survival in patients with primary ovarian cancer.

| | Univariate | | Multivariate | |
|---|---|---|---|---|
| | $RR_1$ | *P*-value | $RR_2$ | *P*-value |
| Stage | | | | |
| I–II | 1[a] | | | |
| III–IV | 2.50 | NS | – | – |
| Grade | | | | |
| 1–2 | 1[a] | | | |
| 3 | 1.67 | NS | – | – |
| Ascites | | | | |
| No | 1[a] | | 1[a] | |
| Yes | 2.01 | <0.05 | 1.59 | <0.05 |
| Residual tumour | | | | |
| ≤2 cm | 1[a] | | | |
| >2 cm | 1.40 | NS | – | – |
| p21 status | | | | |
| ≤2.20 i.a. | 1[a] | | 1[a] | |
| >2.20 i.a. | 2.20 | <0.05 | 1.43 | <0.05 |
| EGFR status | | | | |
| ≤1.5 fmol | 1[a] | | 1[a] | |
| >1.5 fmol | 2.96 | <0.01 | 1.80 | <0.01 |

[a]Reference category. $RR_1$ unadjusted relative risk; $RR_2$ adjusted relative risk, taking into account all the factors of the table. NS, not significant.

ras expression is inversely correlated with the expression of the NM-23 protein, which is believed to play an important role in the suppression of metastases (Scambia et al, 1996).

Our results partly differ from two previous immunohistochemical studies (Rodenburg et al, 1988; Yaginuma et al, 1992) that found no correlation between p21 levels and clinical outcome but are in agreement with recent work by Katsaros et al (1995), performed by immunoblot analysis. This discrepancy may be due to the different methods employed and the use of different monoclonal antibodies – RAP-5 (Rodenburg et al, 1988) and RAP-28 (Yaginuma et al, 1992) – from the one (Y13-259) used both in our study and in that of Katsaros. RAP-5 binds to proteins of a variety of molecular weights in many different neoplastic and normal cell types, while Y13-259 does not recognize ras-related proteins and is of proven specificity to p21 ras (Robinson et al, 1986). RAP-28 recognizes epitopes shared by H-*ras* and K-*ras* gene products, but not by N-*ras*-coded p21, whereas Y13-259 recognizes the epitope shared by all the activated *ras* gene family (Kuzumaki et al, 1986). It is also worth noting that Yaginuma's study (1992) included only 29 patients, 14 of which were at stage I disease. The survival of these early stage patients was only 50% at 5 years, which would suggest that staging procedures were incomplete.

Although in other malignant neoplasms K-*ras* mutations are relatively frequent (Forrester et al, 1987; Liu et al, 1987; Bos, 1989) our data indicate that they are a rare event in ovarian carcinomas. This finding is in agreement with previous studies reporting a combined incidence of K-*ras* mutations of 4–14% in ovarian carcinomas (van't Veer et al, 1988; Enomoto et al, 1990, 1991; Teneriello et al, 1993). The frequency of K-*ras* mutations is particularly low in the most frequent serous histotype. On the other hand, even in the small series analysed, K-*ras* mutations are a frequent event in the less common mucinous carcinomas and in benign and LMP mucinous tumours. This finding, which is in line with previously reported data (Enomoto et al, 1990; Mok et al, 1993; Teneriello et al, 1993; Cuatrecasas et al, 1996), strongly suggests that K-*ras* activation plays an essential role as an early genetic alteration in the pathogenesis of mucinous ovarian tumours irrespective of their malignancy. The overall low incidence of ras mutations and ras amplification (van't Veer et al, 1988; Boltz et al, 1989) and the lack of correlation between ras overexpression and genomic instability (evaluated as aneuploidy) do not account for the high frequency of ras/p21 overexpression that we observed. Further investigations on the mechanism responsible for ras 21 overexpression will be required to address this issue.

It has been reported (Satoh et al, 1990; Dickson et al, 1987) that ras/p21 is involved in the intracellular pathway through which the EGF mitogenic signal is transduced. Moreover, high levels of EGFR are associated with a particularly poor prognosis in ovarian cancer (Scambia et al, 1992; Bartlett et al, 1996). Our data show that both *ras* overexpression and EGFR are independent prognostic parameters. Therefore, it is possible that the simultaneous evaluation of both EGFR and ras may provide complementary information about the presence of alterations of the ras/p21 pathway at different steps, thus improving the prognostic characterization of ovarian cancer patients.

In conclusion, the data presented in this study suggest that the assessment of p21 status at the time of initial surgery may allow the identification of a subset of patients with a particularly poor prognosis. High-risk patients could be candidates for more aggressive and/or experimental primary treatment than that conventionally used. Furthermore, the prognostic significance of p21 levels might imply that ovarian cancer is a candidate for a novel anticancer therapy based on drugs targeted directly against ras activity. Progress has already been made in the development of farnesyltransferase inhibitors which block ras farnesylation and abolish ras-transforming activity 'in vitro' (Gibbs et al, 1994) and the growth of ras-dependent tumours in nude mice (Kohl et al, 1994). Therefore, it would be of interest to test such compounds in patients with ovarian carcinoma overexpressing ras/p21.

### ACKNOWLEDGEMENTS

We thank Massimiliano Marciano for revising the statistical section of the work. This work was partly supported by AIRC (Italian Association for Cancer Research).

### REFERENCES

Bartlett JMS, Langdon SP, Simpson BJB, Stewart M, Katsaros D, Sismondi P, Love S, Scott WN, Williams ARW, Lesseis AM, MacLeod KG, Smyth JF and Miller WR (1996) The prognostic value of epidermal growth factor receptor mRna expression in primary ovarian cancer. *Br J Cancer* **73**: 301-306

Benedetti Panici P, Scambia G, Greggi S, Salerno G, Cento R and Mancuso S (1990) Doxorubicin and cyclophosphamide, alternate with bleomycin and mytomycin C as a second line regimen in advanced ovarian carcinoma resistant to cisplatin based chemotherapy. *Oncology* **47**: 296–298

Benedetti Panici P, Scambia G, Baiocchi G, Greggi S and Mancuso S (1991) Technique and feasibility of systematic paraaortic and pelvic lymphadenectomy for gynecological malignancies: a prospective study. *Int J Gynecol Cancer* **1**: 133–140

Benedetti Panici P, Greggi S, Scambia G, Baiocchi G, Lomonaco M, Conti G and Mancuso S (1993) Efficacy and toxicity of very high-dose cisplatin in

© Cancer Research Campaign 1997

advanced ovarian carcinoma: 4-year survival analysis and neurological follow-up. *Int J Gynecol Cancer* **3**: 44–53

Bland KI, Konstadoulakis MM, Vezeridis MP and Wanebo HJ (1995) Oncogene protein co-expression. Value of *Ha-ras*, *c-myc*, *c-fos*, and *p53* as prognostic discriminants for breast carcinomas. *Ann Surg* **221**: 706–720

Boltz EM, Kefford RF, Leary JA, Houghton CR and Friedlander ML (1989) Amplification of *c-ras-ki* oncogene in human ovarian tumours. *Int J Cancer* **43**: 428–430

Bos JL (1989) ras oncogenes in human cancer: a review. *Cancer Res* **49**: 4682–4689

Bradford M (1976) A rapid and sensitive method for the quantitation of microgram quantities of protein utilising the principle of protein dye-binding. *Anal Biochem* **72**: 248–256

Caulin C, Lopez-Barcons L, Gonzales-Garrigues M, Navarro P, Lozano E, Rodrigo I, Gamallo C, Cano A, Fabra A and Quintanilla M (1996) Suppression of the metastatic phenotype of a mouse skin carcinoma cell line independent of E-cadherin expression and correlated with reduced *H-ras* oncogene products. *Mol Carcinog* **15**: 104–114

Copeland LJ and Gershenson DM (1986) Ovarian cancer recurrences in patients with no macroscopic tumor at second-look laparotomy. *Obstet Gynecol* **68**: 873–874

Cox DR (1972) Regression models and life tables. *J R Stat Soc* **34**: 197–220

Cuatrecasas M, Matias-Guiu X and Prat J (1996) Synchronous mucinous tumors of the appendix and the ovary associated with pseudomyxoma peritonei. A clinicopathologic study of six cases with comparative analysis of *Kras* mutations. *Am J Surg Pathol* **20**: 739–746

Dickson RB, Kasid A, Huff KK, Bates SE, Knabbe C, Bronzert D, Gelmann EP and Lipmann ME (1987) Activation of growth factor secretion in tumorigenic states of breast cancer induced by 17-β-estradiol or v-Ha-*ras* oncogene. *Proc Natl Acad Sci USA* **84**: 837–841

Egan SE, McClarty GA, Jarolim L, Wright JA, Spiro I, Hager G and Greenberg AH (1987) Expression of *Hras* correlates with metastatic potential: evidence for direct regulation of the metastatic phenotype in 10 T1/2 and NIH 3T3 cells. *Mol Cell Biol* **7**: 830–837

Egan SE, Broere JJ, Jarolim L, Wright JA and Greenberg AH (1989) Coregulation of metastatic and transforming activity of normal and mutant ras genes. *Int J Cancer* **43**: 443–448

Enomoto T, Inoue M, Perantoni AO, Terakawa N, Tanizawa O and Rice JM (1990) *K-ras* activation in neoplasms of human female reproductive tract. *Cancer Res* **50**: 6139–6145

Enomoto T, Weghorst CM, Inoue M, Tanizawa O and Rice JM (1991) K-*ras* activation occurs frequently in mucinous adenocarcinomas and rarely in other common epithelial tumors of the human ovary. *Am J Pathol* **139**: 777–785

Forrester K, Almoguera C, Han K, Grizzle WE and Perucho M (1987) Detection of high incidence of K-*ras* oncogenes during human colon tumorigenesis. *Nature* **327**: 298–303

Garbisa S, Pozzatti R, Muschel RJ, Saffiotti U, Ballin M, Goldfarb RH, Khoury G and Liotta LA (1987) Secretion of type IV collagenolytic protease and metastatic phenotype: induction by transfection with c-Ha-ras but not c-Ha-ras plus Ad2-Ela. *Cancer Res* **47**: 1523–1528

Giai M, Roagna R, Ponzone R, De Bortoli M, Dati C and Sismondi P (1994) Prognostic and predictive relevance of c-erbB-2 and ras expression in node positive and negative breast cancer. *Anticancer Res* **14**(3B): 1441–1450

Gibbs JB, Oliff A and Kohl NE (1994) Farnesyltransferase inhibitors: *ras* research yields a potential cancer therapeutic. *Cell* **77**: 175–178

Hanley JA and McNeil BJ (1982) The meaning and the use of the area under a receiver operating characteristic (ROC) curve. *Radiology* **143**: 29–36

Kaplan E and Meier P (1958) Non parametric estimation from incomplete observation. *J Am Stat Assoc* **53**: 457–481

Katsaros D, Theillet C, Zola P, Louason G, Sanfilippo B, Isaia E, Arisio R, Giardina G and Sismondi P (1995) Concurrent abnormal expression of *Erb-b2*, *myc* and *ras* genes is associated with poor outcome of ovarian cancer patients. *Anticancer Res* **15**: 1501–1510

Kohl NE, Wilson FR, Mosser SD, Giuliani E, DeSolms SJ, Conner MW, Anthony NJ, Holtz WJ, Gomez RP, Lee TJ, Smith RL, Graham SL, Hartman GD, Gibbs JB and Oliff A (1994) Protein farnesyltransferase inhibitors block the growth of ras dependent tumors in nude mice. *Proc Natl Acad Sci USA* **91**: 9141–9145

Kuzumaki N, Oda A, Yamagiwa S, Taniguchi N, Kobayashi H, and Oikawa T (1986) Establishment of four mouse hybridoma cell lines producing monoclonal antibodies reactive with ras oncogene product p21. *J Natl Cancer Inst* **77**: 1273–1279

Liu E, Hjelle B, Morgan R, Hecht F and Bishop M (1987) Mutation of the Kirsten-*ras* proto-oncogene in human preleukemia. *Nature* **300**: 186–188

Mantel N (1966) Evaluation of survival data and two new rank order statistics arising in its considerations. *Cancer Chemother Rep* **50**: 163–170

Mizuuchi H, Nasim S, Kudo R, Silverberg SG, Greenhouse S and Garrett CT (1992) Clinical implications of *Kras* mutations in malignant epithelial tumors of the endometrium. *Cancer Res* **52**: 2777–2781

Mok SC-H, Bell DA, Knapp RC, Fishbaugh PM, Welch WR, Muto MG, Berkowitz RS and Tsao SW (1993) Mutation of K-*ras* protooncogene in human ovarian epithelial tumors of borderline malignancy. *Cancer Res* **53**: 1489–1492

O'Brien TJ, Bannon GA, Bard DS, Hardin JW and Quirk JG (1989) Expression of the *ras* oncogene in gynecologic tumors. *Am J Obstet Gynecol* **160**: 344–352

Robinson A, Williams ARW, Piris J, Spandidos DA and Wyllie AH (1986) Evaluation of a monoclonal antibody to *ras* peptide, RAP-5, claimed to bind preferentially to cells of infiltrating carcinomas. *Br J Cancer* **54**: 877–883

Rodenburg CJ, Koelma IA, Nap M and Jan Fleuren G (1988) Immunohistochemical detection of the ras oncogene product p21 in advanced ovarian cancer. *Arch Pathol Lab Med* **112**: 151–154

Rosell R, Li S, Skacel Z, Mate JL, Maestre J, Canela M, Tolosa E, Armengol P, Barnadas A and Ariza A (1993) Prognostic impact of mutated *Kras* gene in surgically resected non-small cell lung cancer patients. *Oncogene* **8**: 2407–2412

Sambrook J, Fritsch EF and Maniatis T (1989) *Molecular Cloning* Vol. 2, Cold Spring Harbor Laboratory Press: Cold Spring Harbor, NY 9.16–9.18

Satoh T, Endo M, Nakafuku M, Akiyama T, Yamamoto T and Kaziro Y (1990) Accumulation of $p21^{ras}$.GTP in response to stimulation with epidermal growth factor and oncogene products with tyrosine kinase activity. *Proc Natl Acad Sci USA* **87**: 7926–7929

Scambia G, Benedetti Panici P, Battaglia F, Ferrandina G, Baiocchi G, Greggi S, De Vincenzo R and Mancuso S (1992) Significance of epidermal growth factor receptor in advanced ovarian cancer. *J Clin Oncol* **10**: 529–535

Scambia G, Catozzi L, Benedetti Panici P, Ferrandina G, Coronetta F, Barozzi R, Baiocchi G, Uccelli L, Piffanelli A and Mancuso S (1993*a*) Expression of *ras* oncogene p21 protein in normal and neoplastic ovarian tissues: correlation with histopathologic features and receptors for estrogen, progesterone and epidermal growth factor. *Am J Obstet Gynecol* **168**: 71–78

Scambia G, Benedetti Panici P, Ferrandina G, Battaglia F, Baiocchi G, Di Stefano P, Tinari N, Coronetta F, Piantelli M, Natali P, Iacobelli S and Mancuso S (1993*b*) Expression of HER-2/neu oncoprotein, DNA ploidy and S-phase fraction in advanced ovarian cancer. *Int J Gynecol Cancer* **3**: 271–278

Scambia G, Benedetti Panici P, Ferrandina G, Distefano M, Romanini ME, Fagotti A and Mancuso S (1995) Epidermal growth factor, oestrogen and progesterone receptor expression in primary ovarian cancer: correlation with clinical outcome and response to chemotherapy. *Br J Cancer* **72**: 361–366

Scambia G, Ferrandina G, Marone M, Benedetti Panici P, Giannitelli C, Piantelli M, Leone A and Mancuso S (1996) NM 23 in ovarian cancer: correlation with clinical outcome and other clinicopathological and biochemical prognostic factors. *J Clin Oncol* **14**: 334–342

Slamon DJ, De Kernion JB, Verma IM and Cline MJ (1984) Expression of cellular oncogenes in human malignancies. *Science* **224**: 256–262

Teneriello MG, Ebina M, Linnoila RI, Henry M, Nash JD, Park RC and Birrer MJ (1993) *p53* and *Ki-ras* mutations in epithelial ovarian neoplasms. *Cancer Res* **53**: 3103–3108

Thorgeirsson UP, Turpeenniemi-Hujanen T, Williams JE, Westin EH, Heilman CA, Talmadge JE and Liotta LA (1985) NIH/3T3 cells transfected with human tumor DNA containing activated *ras* oncogenes express the metastic phenotype in nude mice. *Mol Cell Biol* **5**: 259–262

Van't Veer LJ, Hermens R, Van Den Berg-Bakker LAM, Cheng NC, Fleuren GJ, Bos JL, Cleton FJ and Schrier PI (1988) Ras oncogene activation in human ovarian carcinoma. *Oncogene* **2**: 157–165

Wood WI, Gitschier J, Lasky LA and Lawn RM (1985) Base composition-independent hybridization in tetramethylammonium chloride: a method for oligonucleotide screening of highly complex gene libraries. *Proc Natl Acad Sci* **82**: 1585–1588

World Health Organization (1979) WHO handbook for reporting results of Cancer Treatment, Geneva, Switzerland. WHO 48: 16–21

Yaginuma Y, Yamashita K, Kuzumaki N, Fujita M and Shimizu T (1992) *Ras* oncogene product p21 expression and prognosis of human ovarian tumors. *Gynecol Oncol* **46**: 45–50

© Cancer Research Campaign 1997